# Physicomechanical properties of PMMA denture base resins reinforced with nanofibers: a systematic review and meta-analysis

**DOI:** 10.1590/1678-7765-2026-0076

**Published:** 2026-08-03

**Authors:** Bianca Tavares Rangel, Amanda Aparecida Maia Neves Garcia, Carolina Yoshi Campos Sugio, Vanessa Migliorini Urban, Simone Soares, Joel Ferreira Santiago, André Ulisses Dantas Batista, Karin Hermana Neppelenbroek

**Affiliations:** 1 Universidade de São Paulo Faculdade de Odontologia de Bauru Departamento de Prótese e Periodontia Bauru SP Brasil Universidade de São Paulo, Faculdade de Odontologia de Bauru, Departamento de Prótese e Periodontia, Bauru, SP, Brasil; 2 Universidade Federal de Uberlândia Faculdade de Odontologia Departamento de Prótese Removível e Materiais Dentários Uberlândia MG Brasil Universidade Federal de Uberlândia, Faculdade de Odontologia, Departamento de Prótese Removível e Materiais Dentários, Uberlândia, MG, Brasil; 3 Universidade Estadual de Ponta Grossa Departamento de Odontologia Ponta Grossa Paraná Brasil Universidade Estadual de Ponta Grossa, Departamento de Odontologia, Ponta Grossa, Paraná, Brasil; 4 Universidade de São Paulo Faculdade de Odontologia de Ribeirão Preto Departamento de Materiais Dentários e Prótese Ribeirão Preto SP Brasil Universidade de São Paulo, Faculdade de Odontologia de Ribeirão Preto, Departamento de Materiais Dentários e Prótese, Ribeirão Preto, SP, Brasil; 5 Universidade Federal da Paraíba Departamento de Odontologia Restauradora João Pessoa PB Brasil Universidade Federal da Paraíba, Departamento de Odontologia Restauradora, João Pessoa, PB, Brasil.

**Keywords:** Dentures, Polymethyl methacrylate, Acrylic resins, Nanofibers, Flexural strength

## Abstract

Despite the potential of nanofibers to reinforce poly(methyl methacrylate) (PMMA) denture base resins, no consensus exists regarding their effects on material properties. This systematic review and meta-analysis aimed to evaluate whether the incorporation of nanofibers into PMMA denture base resins improves flexural strength and other physicomechanical properties when compared with unmodified PMMA.

**Methodology:**

This study followed PRISMA guidelines and was registered on the Open Science Framework (<osf.io/3g9pn>). Searches were conducted on Medline/PubMed, Scopus, Cochrane Library, Web of Science, EMBASE, Lilacs, and SciELO. Eligibility criteria included *in vitro* or *in vivo* studies comparing nanofiber-reinforced PMMA to conventional PMMA. The meta-analysis was conducted for flexural strength (the only property assessed in all studies). A subgroup meta-analysis was performed to explore heterogeneity between studies.

**Results:**

From the 1,893 initially screened articles, six were included. The investigated nanofibers included carbon, acrylonitrile-butadiene-styrene, polyamide-6, polystyrene, cellulose, PMMA–cerium, and polyvinyl alcohol at concentrations from 0.1 to 23%. The evaluated properties included impact strength, flexural strength, fracture resistance, hardness, elasticity, toughness, surface roughness, contact angle, and spectrophotometric color analysis. While the qualitative analysis showed divergent findings regarding property improvement, the quantitative analysis revealed no significant differences in flexural strength between nanofiber-reinforced PMMA and conventional PMMA at the evaluated concentrations: PMMA *vs*. 0.5% PMMA (p=0.554), PMMA vs. 1% PMMA (p=0.847), PMMA vs. 3% PMMA (p=0.9825), and PMMA vs. 5% PMMA (p=0.222). However, subgroup analysis indicated higher flexural strength for the conventional PMMA when compared with nanofiber-reinforced PMMA, particularly at higher nanofiber concentrations (p<0.0001).

**Conclusion:**

Incorporating nanofibers into PMMA resulted in no significant increase in flexural strength, showing inconsistent effects on other physicomechanical properties. Therefore, further standardized laboratory and clinical studies are needed to clarify its efficacy and clinical applicability.

## Graphical abstract

**Figure:**
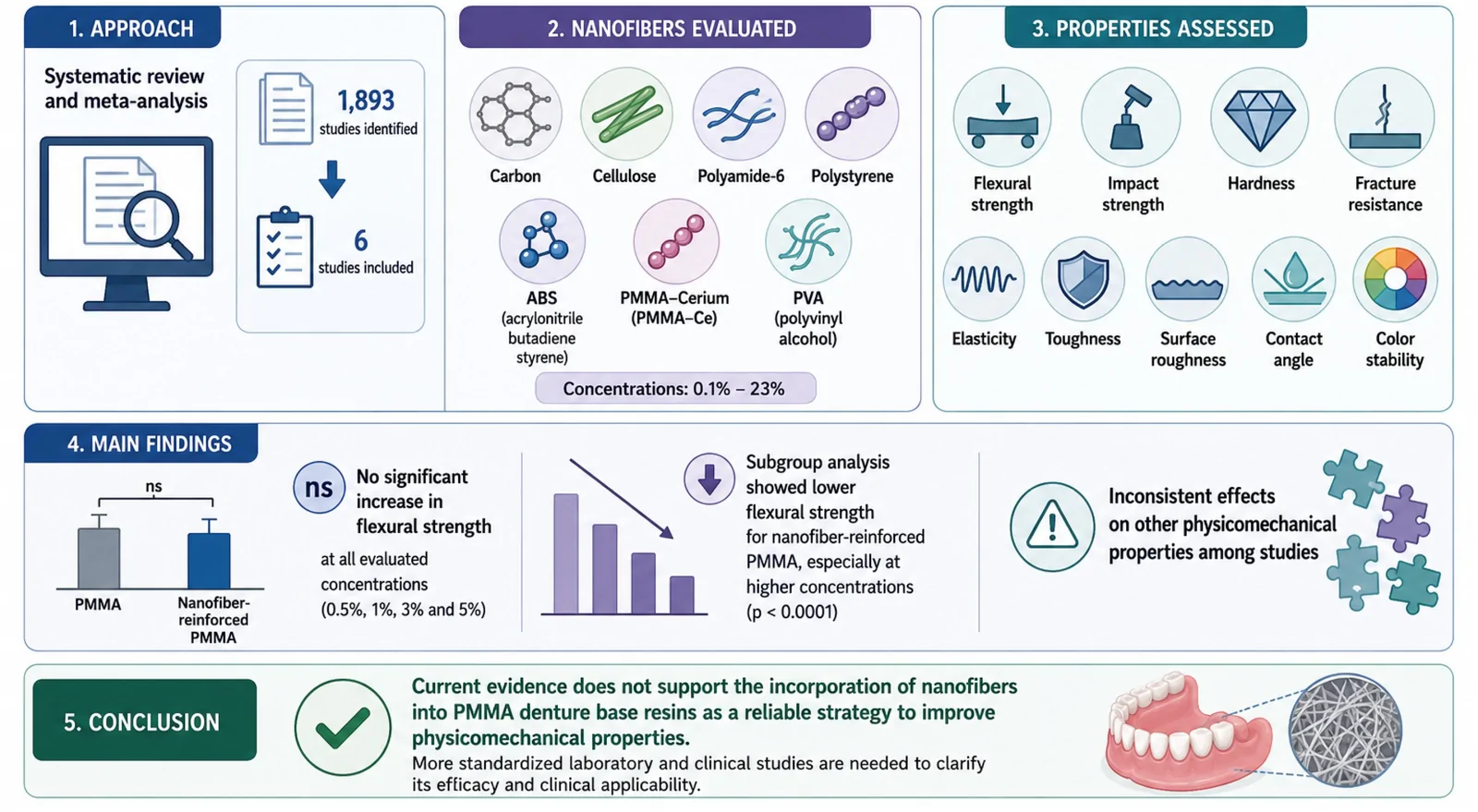

## Introduction

Poly(methyl methacrylate) (PMMA) remains the most widely used material for fabricating acrylic bases in complete and partial removable dentures due to its favorable properties such as ease of processing and polishing, stability in the oral environment, excellent optical characteristics, and relatively low cost.^1,2^ However, it has limited mechanical performance, particularly regarding flexural and impact strength.^3^ Dimensional changes from polymerization shrinkage may generate residual stresses that increase the susceptibility of the material to crack initiation and propagation, especially under cyclic masticatory loading.^4^ Moreover, microporosities resulting from monomer evaporation, insufficient packing pressure, or air entrapment may act as stress concentrators and further weaken the material.^5^ These factors contribute to the reduced mechanical reliability of PMMA denture base resins.^6^ Fractures remain clinically common, particularly in maxillary complete dentures,^7^ and may result in patient discomfort, repair or replacement needs, and potential health risks associated with aspiration or ingestion of fractured fragments.^8,9^

Given these limitations, reinforcement strategies via the incorporation of nanoparticles, microscale fibers, and nanofibers (NFs) have been investigated to improve the physicomechanical performance of PMMA^1,10,11^ and reduce denture fractures.^5^ NFs are typically defined as fibers with diameters below 100 nm and high aspect ratios, exhibiting predominantly one-dimensional structural characteristics.^12^ Due to their high surface area-to-volume ratio and tunable architecture, NFs have been associated with improved flexural strength, impact resistance, tensile strength, and interlaminar shear resistance.^13-15^ Unlike particulate fillers, NFs may be distributed and oriented within the polymer matrix,^16^ improving load transfer at the fiber–matrix interface and promoting microcrack bridging under masticatory loading.^17^ Although nanoparticles and microscale fibers have also been investigated, limitations related to biological safety, homogeneous dispersion, and stress concentration have been reported.^5,18,19^ Therefore, NFs represent a promising reinforcement strategy for PMMA denture base resins due to their potential to improve stress distribution and fracture resistance within the polymer matrix.^5,17,20^

In this context, several types of fibers, including nylon, polyethylene, polyamide, and glass fibers, have been investigated as reinforcing agents for PMMA denture base resins.^21,22^ Their incorporation aims to improve strength, stiffness, and impact resistance, reducing fracture susceptibility and potential overload on supporting structures.^5,23,24^

Despite these potential benefits, only one previous systematic review has evaluated reinforcing agents for denture base resins, including heterogeneous materials such as metallic particles, metal or ceramic oxides, glass fibers, and nanotubes.^25^ This heterogeneity limited direct comparison and evidence synthesis.^25^ Thus, to date, no systematic review and meta-analysis has evaluated the influence of NF reinforcement on the physicomechanical properties of PMMA denture base resins. Given the potential clinical relevance of improving PMMA performance for removable denture users, this systematic review aimed to evaluate whether the incorporation of NFs into PMMA denture base resins improves flexural strength and other physicomechanical properties when compared with unmodified PMMA.

## Methodology

This study was conducted in accordance with the methodological criteria for systematic reviews and meta-analyses established by the Cochrane Collaboration (Cochrane Handbook for Systematic Reviews of Interventions – Handbook 5.1.0) and the Preferred Reporting Items for Systematic Reviews and Meta-Analyses (PRISMA 2020) statement. The protocol was registered on the Open Science Framework on November 18, 2023 (<osf.io/3g9pn>).

This study was structured around the following research question: Can NFs improve the physicomechanical properties of PMMA denture base resins? The analysis followed the PICO strategy: population (P) — PMMA denture base resins; intervention (I) — PMMA reinforced with NFs; comparison (C) — conventional PMMA; and outcomes (O) — flexural strength (primary outcome) and other physicomechanical properties.

The electronic databases Medline/PubMed, Scopus, Web of Science, EMBASE (Excerpta Medica Database), SciELO (Scientific Electronic Library Online), and LILACS (Latin American and Caribbean Health Sciences Literature) were systematically searched up to May 25, 2026. Search strategies were tailored for each database by combining controlled vocabulary terms (MeSH for PubMed, Emtree for EMBASE, and DeCS for LILACS/SciELO) with free-text keywords and their variations. For databases without a controlled vocabulary, such as Scopus and Web of Science, search strategies were developed using comprehensive combinations of free-text terms, synonyms, and truncations. The search terms included combinations of the following: ("PMMA" OR "poly(methyl methacrylate)" OR "acrylic resin" OR "denture base" OR "complete denture" OR "denture base resin") AND ("nanofiber" OR "nanofibers" OR "nano-fiber" OR "nanofibre" OR "nanofibres" OR "fiber-reinforced" OR "reinforcement" OR "carbon nanofiber" OR "carbon nanotube fiber" OR "graphene nanofiber" OR "glass nanofiber" OR "silica nanofiber" OR "ceramic nanofiber" OR "polyamide nanofiber" OR "cellulose nanofiber" OR "aramid nanofiber" OR "polystyrene nanofiber" OR "polyacrylonitrile nanofiber" OR "polyurethane nanofiber" OR "polyvinyl alcohol nanofiber" OR "polymeric nanofiber" OR "PMMA nanofiber"). Boolean operators (AND, OR) were used to combine terms.

The inclusion criteria comprised clinical and *in vitro* studies that evaluated PMMA denture base resins modified with NFs and included at least one control group (conventional PMMA denture base resin). Studies that failed to evaluate PMMA denture base resins and/or NF incorporation and those using other nanostructures were excluded from this review. Case reports, opinion papers, correspondences, editorials, and letters to the editor were also excluded. Articles were managed (including duplicate removal) on EndNote 20 (Clarivate, Philadelphia, PA, USA). No clinical studies meeting the eligibility criteria were identified. Therefore, only *in vitro* studies were included in the final analysis.

Then, two reviewers (AAMNG and BTR) independently screened titles and abstracts according to the eligibility criteria. Prior to formal selection, a calibration was performed using a sample of studies to ensure consistency between reviewers. Inter-reviewer agreement was assessed using Cohen's kappa coefficient, which indicated almost perfect agreement (κ= 0.84). Discrepancies were resolved via discussion, and when necessary, a third reviewer was consulted to reach consensus (JFSJr). Clinical and scientific guidance for data extraction and interpretation was provided by researchers with expertise in acrylic denture base resins (KHN and VMU) and NFs (AUDB).

The following data were extracted from each study: (1) author, publication year, and country; (2) PMMA denture base material and polymerization method; (3) NF characteristics; (4) NF production method; (5) PMMA reinforcement method using NFs; (6) sample size (n), sample dimensions, and followed standards; (7) evaluated physicomechanical properties; (8) percentage by weight of NF used for PMMA reinforcement; (9) reported outcomes (percentages, means, and p-values); and (10) main conclusion. Data were recorded on a standardized Excel spreadsheet for final data tabulation. Missing data were reported as "not specified (N/S)," and unclear information was recorded as "unclear." Due to substantial methodological variability between studies, the meta-analysis was restricted to flexural strength as it was the only consistently reported outcome.

Risk of bias was assessed using criteria adapted from tools for clinical studies to suit *in vitro* investigations on RevMan (version 5.3; The Nordic Cochrane Centre, Copenhagen, Denmark). The assessment considered methodological reporting clarity, adherence to experimental standards, presence of control groups, and adequacy of outcome reporting. NF characterization was only evaluated when performed prior to incorporation into the PMMA matrix. Moreover, a critical appraisal of the study design, measurement methods, and consistency between reported results and conclusions was conducted. Based on the overall qualitative assessment of these domains, studies were classified as having low, moderate, or high risk of bias.

Quantitative data were analyzed for flexural strength using mean values, standard deviations, and sample sizes. Continuous data were analyzed using mean differences (MD), 95% confidence intervals (CI), and a p<0.05 significance level. Forest plots were generated on MetaAnalysisOnline (A5 Genetics Ltd; available at https://metaanalysisonline.com/).^26-29^

Flexural strength was selected as the primary outcome because it is a clinically relevant property for denture base materials, is addressed in the International Organization for Standardization (ISO) 20795-1 requirements (65 MPa minimum flexural strength),^30^ and was the only outcome consistently reported in all included studies. Additionally, group differences were interpreted based on statistical significance between NF-reinforced PMMA and control groups. Heterogeneity was assessed using the Q test and the I^2^ index.^27^ I^2^ values above 75% (on a scale of 0 to 100%) were considered indicative of high heterogeneity.^27,31^ A random-effects model was applied in all meta-analyses.^32^ Subgroup analyses were conducted based on NF concentration to explore sources of variability. When substantial heterogeneity persisted, exploratory *post hoc* subgroup analyses were performed to assess the influence of individual studies with distinct methodological characteristics on pooled estimates.

## Results

### Literature search

The search across all databases yielded 1,893 articles. After duplicate removal, 15 articles were screened for eligibility, and six studies were included in the final stage of this systematic review (Figure 1).

**Figure 1:**
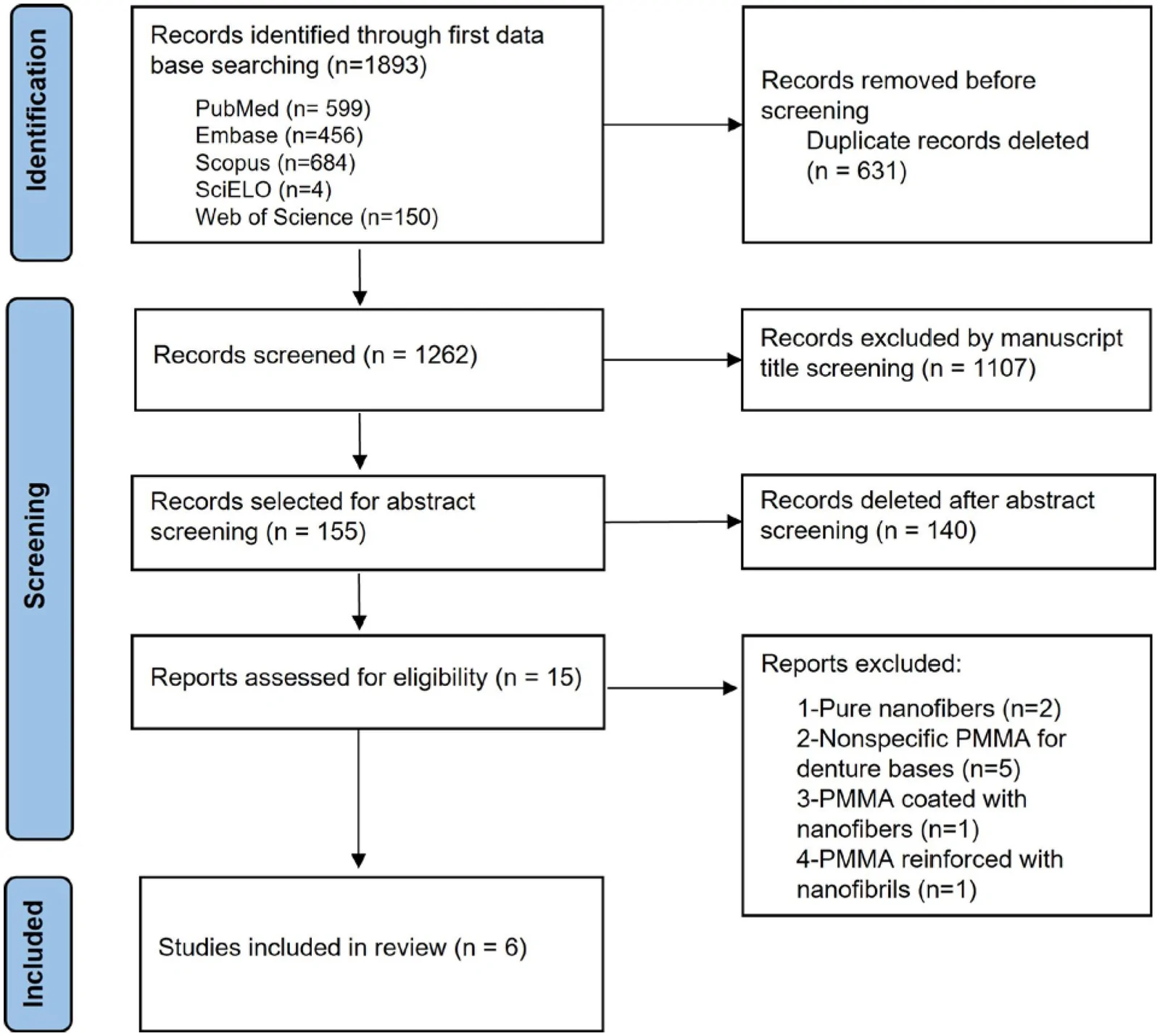
PRISMA flow diagram of the study selection process, detailing the identification, screening, eligibility, and inclusion stages of the systematic review.

### Study characteristics

The selected studies were *in vitro* investigations published from 2016 to 2025 that compared the physicomechanical properties of PMMA denture base resin reinforced with NFs with conventional PMMA. No clinical studies fulfilling the predefined eligibility criteria were identified in the literature. Therefore, only *in vitro* studies were included in the analysis. All included studies comprised more than one experimental group and a control group consisting of conventional autopolymerized, heat-polymerized, or thermoplastic PMMA. The NFs investigated were composed of carbon,^33^ acrylonitrile–butadiene–styrene, polyamide-6, polystyrene,^7^ cellulose,^34,35^ PMMA–cerium (PMMA-Ce),^36^ and polyvinyl alcohol^2^ (Table 1).

**Table 1:** Summary of the methodological characteristics and results of the included studies

Study	Comparisons		Intervention		Settings		Outcome measured		Main conclusion	
Author, publication year, and country	PMMA denture base material/polymerization method	NF details	NF production method	PMMA reinforcement method by NFs	Sample size (n), sample dimensions and standards followed	Physical and Mechanical Tests Performed	NF wt% used for PMMA resin reinforcement	Findings (%, means)		
Dai, et al.^33^ (2021)	Autopolymerizing acrylic	Carbon (CNF)	N/S	CNF powders were mixed with pre-polymerized PMMA powders.	n= 10* Dimensions: 80×10×4.5 mm	Flexural strength (Shimadzu AG-XPLUS10KN, Japan), Flexural modulus, Impact strength (XJU-5.5, Chengde Jinjian Testing Instrument Co., Ltd., China), and hardness (TH300, Beijing TIME High Technology Ltd. China).	Flexural strength (MPa)			CNFs improved the mechanical properties of PMMA-based denture resins.
							Wt%	Mean ±SD		
							Control	48.85 ± 4.62		
							0.5%	66.44 ± 1.40		
							1%	68.75 ± 6.82		
							3%	89.26 ± 1.97		
							Impact strength (kJ m^-2^)			
							Wt%	Mean ±SD		
							Control	5.50 ± 0.42		
							0.5%	31.25 ± 1.42		
							1%	29.11 ± 0.73		
							3%	40.15 ± 1.58		
							Hardness (HRC)			
							Wt%	Mean ± SD		
							Control	65.8 ± 6.71		
							0.5%	79.4 ± 5.11		
							1%	83.1 ± 2.74		
							3%	96.01 ± 5.92		
Gonçalves, et al.^7^ (2020) Brazil	Vipi Wave; Vipi Dental Products, Pirassununga, SP, Brazil/ Polymerized by the microwave: 20 minutes at 10% power and 5 minutes at 60% power	Acrylonitrile-butadiene-styrene (ABS), polyamide-6 (PA6),	Electrospinning	The PMMA was handled and a NF mat was positioned (it was not necessarily incorporated).	n=5 30×10×3 mm Standard: N/S	Flexural strength and fracture resistance (universal testing machine)	2, 3, and 4% for the ABS, P6, and PS groups, respectively. The fiber volume was fixed at ∼15 vol% for all groups.			Despite the lack of direct reinforcement of the mechanical strength of the PMMA resin, there was an improvement in fracture behavior, making it a more compliant material.
							Flexural strength (MPa)			
							NF group	Wt%	Mean ±SD	
							Control	0	111.0 ± 20.0	
							ABS	2%	97.1 ± 4.5	
							P6	3%	85.9 ± 10.1	
							PS	4%	90.7 ± 10.1	
							Fracture resistance (J/m^2^)			
							NF group	Wt%	Mean	
							Control	0	7.5	
							ABS	2%	7.5	
							P6	3%	9.3	
							PS	4%	6.6	
Kawaguchi, et al.^34^ (2020) Japan	Acry Jet, Denken-Highdental Co., Kyoto, Japan/ thermoplastic, injection- molded under temperature and pressure	Cellulose (CNF)	N/S	PMMA pellets were mixed with CNF	n=12 64×10±0.2 ×3.3±0.2 mm Standard: adapted ISO	Flexural strength, flexural modulus (Autograph AGS-J, Shi-madzu Corp., Kyoto, Japan)	Flexural strength (MPa)			CNF reinforcement improves the flexural strength and flexural modulus of PMMA denture base materials prepared by injection molding.
							Wt%	Mean ±SD		
							Control	49.4 ±0.7		
							5%	56.4 ±1.3		
							10%	63.5 ±2.0		
							15%	72.0 ±4.7		
							23%	96.8 ±4.0		
							Flexural modulus (GPa)			
							Wt%	Mean ±SD		
							Control	1.31 ±0.02		
							5%	1.56 ±0.05		
							10%	1.99 ±0.14		
							15%	2.40 ±0.15		
							23%	3.96 ±0.08		
Rangel, et al.^36^ (2025) Brazil	Onda-Cryl, Dentsply Sirona York, PA, USA/Polymerized by the microwave: 360 W for 3 min, 0 W for 4 min, and 900 W for 3 min	Polymethyl methacrylate (PMMA) – cerium (Ce)	Solution blow spinning (SBS) technique	The NFs were first blended with the powder, followed by the addition of the liquid.	n=12 65 mm × 10 mm × 4 mm fo flexural strength Testing, and 10 mm × 5 mm for all other physicomechanical Standard: ISO 20795-1 (flexural strength)	Flexural strength (Instron r Co., Norwood, MA, USA), knoop microhardness (MicroMet 6040 – Buehler, Buehler LTD, Lake Bluff, IL, USA), surface roughness (CCI MP, Taylor Hobson, Leicester, Leicestershire, United Kingdom), Contact angle (OCA 15EC, Filderstadt, Freiburg, Ger many), spectrophotometric analysis of color (Delta Color, São Leopoldo, RS, Brazil)	Flexural strength (MPa)			The incorporation of PMMA- Ce NFs impaired the physicomechanical properties of the acrylic resin, reducing flexural strength and microhardness, increasing the contact angle, and causing color changes, while surface roughness remained unaffected.
							Wt%	Mean ±SD		
							Control	98.50 ± 10.96		
							0.5%	93.60 ± 8.67		
							1%	86.20 ± 10.62		
							2.5%	84.10 ± 11.28		
							5%	78.20 ± 13.17		
							Knoop Microhardness (KHN)			
							Wt%	Medians ± IQ		
							Control	18.20 ± (17.90–18.20)		
							0.5%	16.60 ± (16.30–17.50)		
							1%	17.20 ± (16.90–17.50)		
							2.5%	17.20 ± (16.80–17.60)		
							5%	16.10 ± (15.80–16.70)		
							Surface roughness (Sa) (μm)			
							Wt%	Medians ± IQ		
							Control	0.14 ± (0.09–0.17)		
							0.5%	0.11 ± (0.09–0.16)		
							1%	0.12 ± (0.10–0.14)		
							2.5%	0.10 ± (0.08–0.15)		
							5%	0.14 ± (0.11–0.15)		
							Surface roughness (Sq) (μm)			
							Wt%	Medians ± IQ		
							Control	0.16 ± (0.13–0.21)		
							0.5%	0.16 ± (0.13–0.21)		
							1%	0.16 ± (0.13–0.23)		
							2.5%	0.16 ± (0.13–0.21)		
							5%	0.19 ± (0.17–0.22)		
							Contact angle (°)			
							Wt%	Mean ±SD		
							Control	72.10 ± 3.10		
							0.5%	80.70 ± 1.88		
							1%	82.10 ± 3.80		
							2.5%	82.20 ± 3.87		
							5%	82.60 ± 13.17		
							Color parameter L*			
							Wt%	Medians ± IQ		
							Control	51.60 ± (51.30–52.2)		
							0.5%	51.00 ± (50.20–51.60)		
							1%	51.30 ± (50.60–52.40)		
							2.5%	53.70 ± (53.00–54.8)		
							5%	53.30 ± (53.00–54.1)		
							Color parameter a*			
							Wt%	Mean ±SD		
							Control	30.30 ± 0.91		
							0.5%	29.80 ± 1.37		
							1%	29.60 ± 1.45		
							2.5%	26.50 ± 1.84		
							5%	26.20 ± 1.93		
							Color parameter b*			
							Wt%	Mean ±SD		
							Control	18.40 ± 0.61		
							0.5%	18.80 ± 0.10		
							1%	19.40 ± 0.52		
							2.5%	17.50 ± 1.56		
							5%	18.20 ± 1.05		
Uyar, et al.^2^ (2016) Turkey	Vertex, Zeist, Holland/thermally activated by boiled water for 20 min	Polyvinyl alcohol (PVA)	Custom electrospinning	The PMMA powder and liquid were mixed and then the NF mat was placed.	n=N/S 64×10×3.3mm Standard: ASTM D790	Flexural strength (Instron 8874, Boston, USA), elastic modulus, and toughness	0.05; 0.25; 1, and 1.25% The authors reported only the maximum values of each test and described the remaining data in graphs. Flexural strength: maximum value 5.1 MPa Elastic modulus: maximum value 0.8 GPa Hardness: maximum value 170 kJ/m^3^ The MTT assay results showed that the PVA-PMMA composite and the control group have similar absorbance levels.			PVA NFs improved the mechanical properties of PMMA dental composites. Moreover, the produced material showed no toxicity.
Yamazaki, et al.^35^ (2020)	Acron; GC, Tokyo, Japan/thermally activated	Cellulose (CNF)	Dehydration of an aqueous CNF solution, pressurization, and drying.	The PMMA powder and liquid were mixed and the NFs were added.	n=5 64×10×3.3mm Standard: ISO 20795-1	Flexural strength and flexural modulus (Instron, Canton, MA, USA, model 5565)	Flexural strength (MPa)			Adding CNF to PMMA reduced the mechanical strength.
							Wt%	Mean ±SD		
							Control	90.32±4.66		
							0.1%	83.79±5.55		
							0.3	75.87±6.33		
							1%	75.82±5.04		
							3%	74.43±4.46		
							5%	64.42±7.32		
							Flexural modulus (MPa)			
							Wt%	Mean ±SD		
							Control	2253.52±135.67		
							0.1%	2142.89±152.86		
							0.3%	2287.03±116.21		
							1%	2453.42±82.38		
							3%	2488.27±93.98		
							5%	2486.47±108.18		

* Sample size information obtained through personal communication with the corresponding authors

### Synthesis of experimental material and specimens

The included studies showed methodological heterogeneity regarding NF production methods and incorporation techniques. The number of specimens ranged from 5 to 12,^2,7,34-36^ and missing information from one study was clarified through correspondence with the corresponding author (n=10).^33^ Variations were also observed in the standards for specimen preparation and in NF concentrations, which ranged from 0.1 to 23% by weight (Table 1).

### Evaluation of mechanical properties and effects of NF addition to PMMA

The evaluated physicomechanical properties included flexural strength,^2,7,33-36^ impact strength,^33^ hardness,^33,36^ fracture resistance,^7^ elasticity,^2^ toughness,^2^ surface roughness, contact angle, and spectrophotometric color analysis.^36^ The only property evaluated by all studies was flexural strength (Table 1).

Adding NFs improved the mechanical properties of PMMA denture base resin in three studies,^2,33,34^ whereas three investigations reported a reduction in mechanical performance, particularly in flexural strength.^7,35,36^

In the study by Dai, et al.^33^ (2021), the incorporation of carbon NFs significantly improved all evaluated mechanical properties, including flexural strength, flexural modulus, impact strength, and hardness, when compared with unmodified PMMA, with the highest values observed at 3% NFs.

Similarly, Kawaguchi, et al.^34^ (2020) reported a significant increase in flexural strength and modulus following NF incorporation into PMMA. These properties tended to improve as the NF content increased, with the highest values obtained at the highest concentrations.

In contrast, Uyar, et al.^2^ (2016) reported neither statistical analyses nor significance values. The authors only described numerical increases in flexural strength, elastic modulus, and toughness of NF-modified PMMA, highlighting the maximum values obtained for NF-reinforced composites (without statistical confirmation).

In the study by Gonçalves, et al.^7^ (2020), NF-modified PMMA groups (ABS and PS) showed numerically lower flexural strength values than the control, with no statistically significant differences. Only the P6 group showed a statistically significant reduction in flexural strength relative to unmodified PMMA.

Similarly, Yamazaki, et al.^35^ (2020) observed a significant reduction in flexural strength across all NF-modified PMMA groups when compared with the control. However, flexural modulus was significantly higher in groups containing 1, 3, and 5% carbon NFs.

Finally, Rangel, et al.^36^ (2025) reported a significant reduction in the flexural strength of PMMA at all tested NF concentrations. Color changes were observed at 2.5 and 5%, and hardness was significantly reduced at 0.5 and 5%. Regardless of concentration, NF incorporation significantly increased the contact angle of PMMA, whereas no statistically significant differences were observed in surface roughness between groups.

### Quantitative Analysis

#### Flexural strength analysis (Control vs. 0.5% Reinforcement)

A total of 22 specimens were included in the analysis of the conventional PMMA group (control) versus the group of PMMA reinforced with 0.5% carbon NF^33^ or PMMA-Ce NF.^36^ The meta-analysis performed with two studies showed no statistically significant difference between the control and 0.5% NF groups (MD: 6.65; 95% CI: −15.38 to 28.69, p=0.5538, I^2^=96.3; Figure 2).

**Figure 2:**
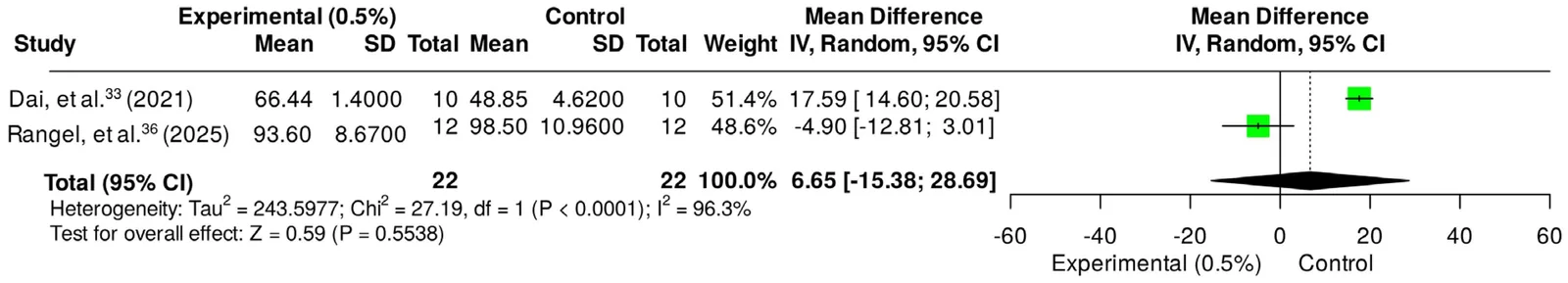
Forest plot of mean difference (MD) between conventional and reinforced PMMA with 0.5% NFs.

#### Flexural strength analysis (Control vs. 1% Reinforcement)

The comparative analysis between the control group and the group of PMMA reinforced with 1% carbon NFs,^33^ PMMA-Ce NF,^36^ or cellulose NFs^35^ found 54 specimens. The meta-analysis with three studies showed no significant differences between groups (MD: −2.16; 95% CI: −24.12 to 19.80, p=0.8471, I^2^=97.7; Figure 3).

**Figure 3:**
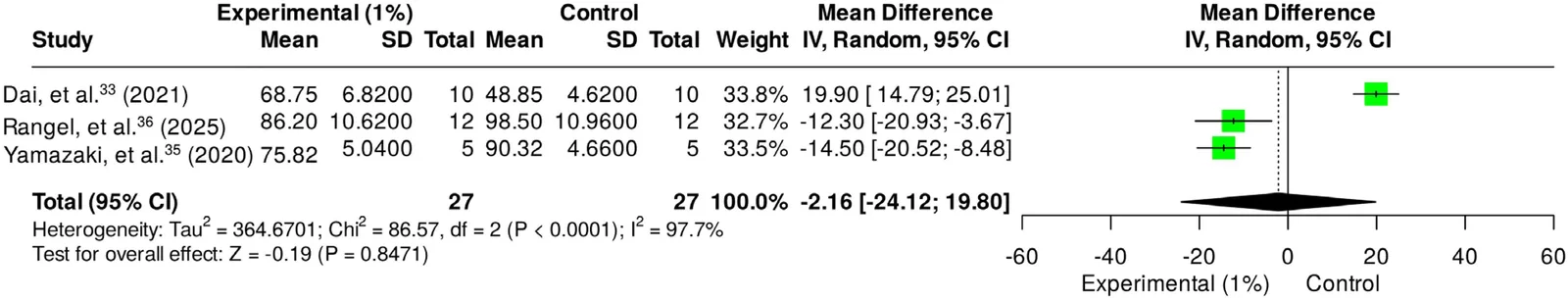
Forest plot of mean difference (MD) between conventional and reinforced PMMA with 1% NFs.

#### Flexural strength analysis (Control vs. 3% Reinforcement)

In the analysis of the control group versus the group with 3% NFs of carbon,^33^ polyamide-6,^7^ and cellulose,^35^ 40 specimens were identified. The meta-analysis with three studies showed no significant difference in the comparison between groups (MD: 0.45; 95% CI: −39.99 to 40.89, p=0.9825, I^2^=99.4; Figure 4).

**Figure 4:**
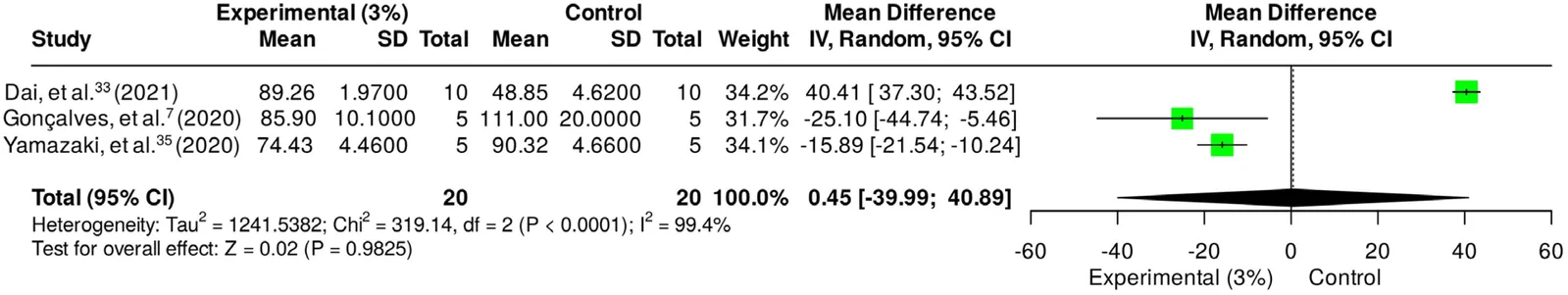
Forest plot of mean difference (MD) between conventional and reinforced PMMA with 3% NFs.

#### Flexural strength analysis (Control vs. 5% Reinforcement)

In the analysis of the control group versus the group with 5% cellulose NFs,^34,35^ and PMMA-Ce NF,^36^ 58 specimens were identified. The meta-analysis with three studies showed no statistically significant difference between the control and 5% NFs groups (MD: −12.68; 95% CI: −33.03 to 7.67, p=0.2219, I^2^=98.0; Figure 5).

**Figure 5:**
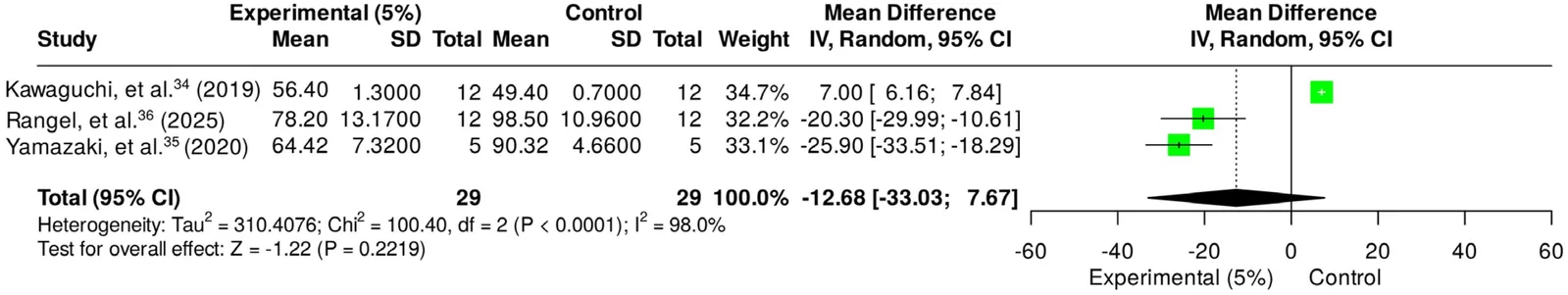
Forest plot of mean difference (MD) between conventional and reinforced PMMA with 5% NFs.

#### Subgroup analysis

The studies by Dai, et al.^33^ (2021) and Kawaguchi, et al.^34^ (2020) were excluded in an exploratory subgroup analysis conducted after the initial meta-analyses. Following this exclusion, three studies remained eligible for quantitative synthesis. Heterogeneity was reduced to 0% (I^2^ = 0%) for comparisons at 1, 3, and 5% NF concentrations *versus* the control group. A total of 112 specimens were included in this subgroup analysis.

The control group showed significantly greater flexural strength than the NF-reinforced groups (p<0.0001), with moderate heterogeneity (I^2^=63.8%) and a mean difference of −16.10 (95% CI: −21.42 to −10.79). Greater differences between control and NF-reinforced groups were observed at higher NF concentrations (p=0.0020) (Figure 6).

**Figure 6:**
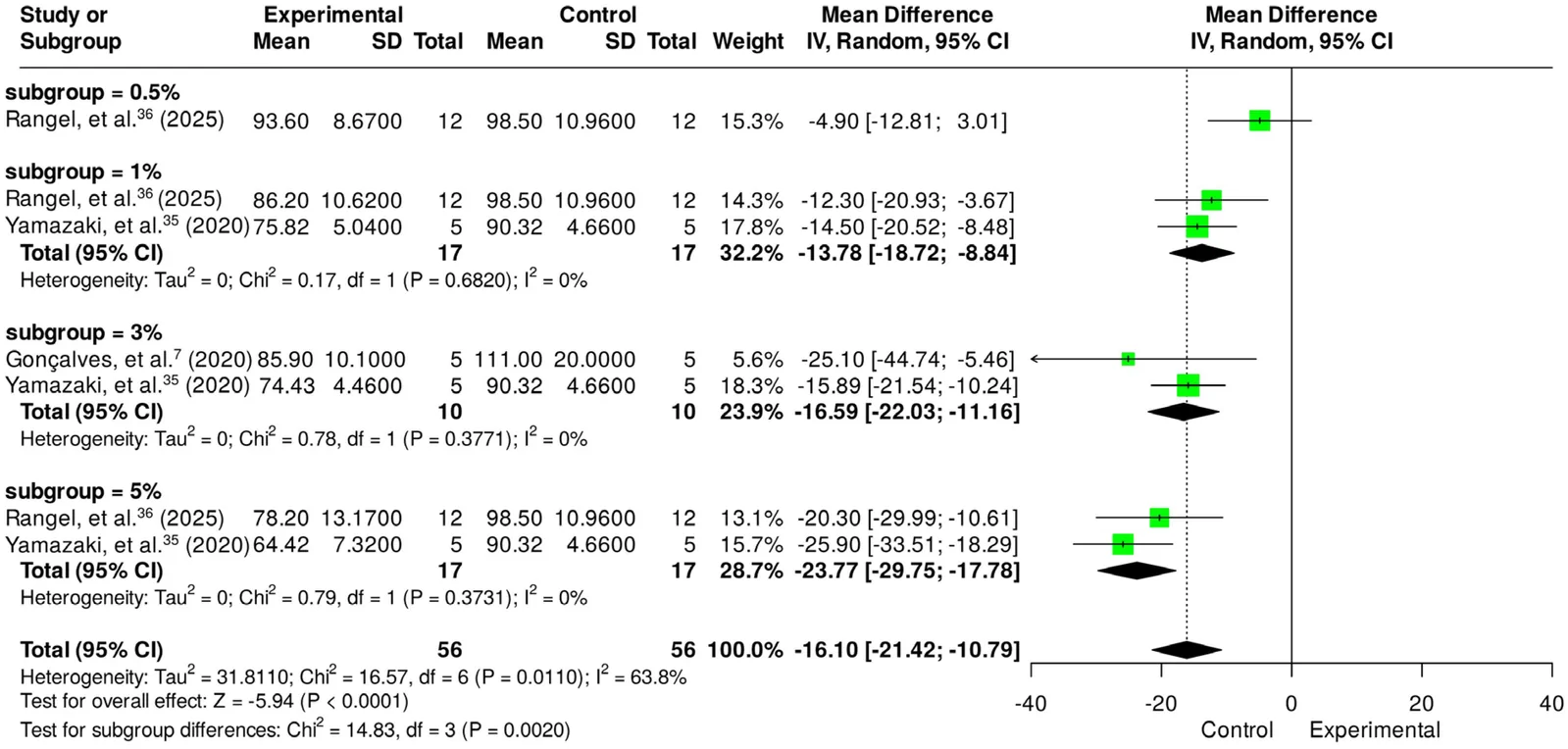
Forest plot of subgroup analysis of mean difference (MD) in flexural strength between conventional PMMA and NFs-reinforced PMMA (1%, 3%, and 5% NFs).

Across the included studies, most experimental and control groups met the minimum flexural strength requirement established by ISO 20795-1 (65 MPa).^30^ However, one study^35^ reported values below this threshold for the group containing 5% NFs (64.42 MPa).

### Risk of bias

The data from the risk of bias analysis are shown in Figure 7. All included studies were classified as having a moderate risk of bias. Regarding the quality of their control groups and conclusions, all selected investigations were considered to have a moderate risk of bias. Regarding their methodological design, the studies were rated as having a moderate risk of bias due to the absence of sample size calculations, which may limit their statistical power. Considering the characterization of material, Dai, et al.^33^ (2021), Kawaguchi, et al.^34^ (2020), and Yamazaki, et al.^35^ (2020) were classified as having a high risk of bias because they performed no specific characterization analyses, which may compromise reproducibility. Regarding methodological evaluation, Dai, et al.^33^ (2021) and Uyar, et al.^2^ (2016) showed a high risk of bias due to the absence of information on sample size and insufficient reporting of statistical methods. These limitations included the lack of normality testing, absence of description and justification of the used statistical tests, and failure to report p-values, which may affect the reliability and robustness of their findings. Finally, regarding the clarity of result reporting, the investigations by Gonçalves, et al.^7^ (2020), Uyar, et al.^2^ (2016), and Yamazaki, et al.^35^ (2020) were considered to have a high risk of bias due to the absence of detailed numerical data, which restricts direct comparison between groups.

**Figure 7:**
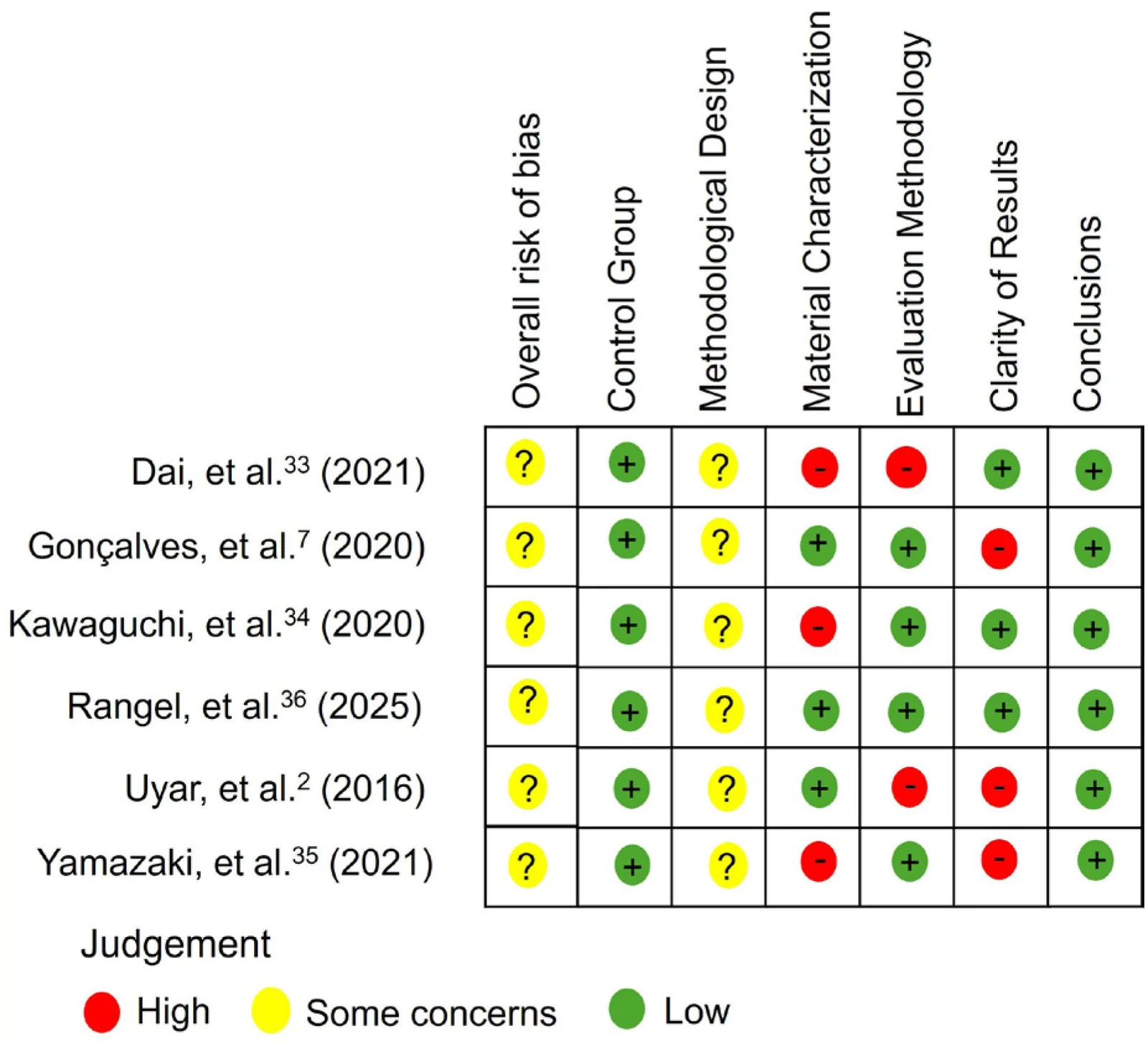
Risk of bias of the included studies.

## Discussion

This systematic review and meta-analysis evaluated whether incorporating NF into PMMA denture base resins improves flexural strength and other physicomechanical properties when compared with conventional PMMA. Overall, the available evidence showed no consistent benefits associated with NF incorporation in relation to conventional PMMA. Although the primary meta-analysis found no statistically significant differences in flexural strength between NF-reinforced and conventional PMMA, the exploratory subgroup analysis suggested that methodological variability substantially influenced the pooled findings. Methodological differences may explain the distinct behavior in the studies by Dai, et al.^33^ (2021) and Kawaguchi, et al.^34^ (2020). The pre-polymerized PMMA powder in the investigation by Dai, et al.^33^ (2021) may have contributed to a reduced amount of residual monomer and, consequently, to improved flexural strength. Monomer–polymer conversion degree and residual monomer content directly influence the physicomechanical properties of acrylic resins.^37,38^ When polymerization is incomplete, residual monomer remains within the polymer matrix,^39^ acting as a plasticizer and potentially compromising these properties.^40^ In contrast, Kawaguchi, et al.^34^ (2020) used a thermoplastic PMMA resin, which differs in structure and processing from conventional acrylic resins. These differences may have influenced the mechanical behavior observed.^41^

These findings may be partially attributed to the limited number of included studies and the resulting small sample size and uncertainty regarding statistical power since no investigation reported sample size calculations. Furthermore, the qualitative analysis of the other evaluated physicomechanical properties showed inconsistent results, with approximately half of the included studies reporting significant improvements after NF incorporation, whereas the remainder observed no beneficial effects. These inconsistencies may also be associated with material-related factors. The effectiveness of fiber reinforcement depends on efficient stress transfer from the polymer matrix to the fibers, which is often enhanced by coupling agents that promote chemical bonding between the filler and the resin matrix.^42^ Other variables that may influence reinforcement effectiveness include resin type, fiber content, interfacial adhesion quality, fiber length, and processing conditions.^43^ Notably, no included study reported using coupling agents, and variability was observed in resin type—including autopolymerized,^33^ heat-polymerized,^2,7,35,36^ and thermoplastic^34^ PMMA—and commercial brands. These factors may have contributed to heterogeneous stress distribution, reduced interfacial bonding efficiency, and, consequently, inconsistent mechanical outcomes.

Additional factors that may affect the mechanical properties of fiber-reinforced resins include the aspect ratio of the NFs and the orientation of the fibers within the polymer matrix.^17^ Processing parameters are also critical for the performance of fiber-reinforced composites as they directly influence the quality of fiber incorporation into the polymer matrix and the formation of structural defects, such as agglomerations and voids, which may compromise mechanical properties due to stress concentration.^44^ These effects, widely reported in composite materials, are also likely to be relevant in PMMA-based denture base resins. Even among studies that used the same type of heat-polymerized PMMA,^2,7,35,36^ methodological differences were observed, including variations in polymerization procedures, such as the use of boiling water^2^ or microwave energy^7,36^ under different conditions. These differences may also have contributed to the variability in the reported results.

Flexural strength was selected as the primary outcome because it was the only outcome consistently reported across all included studies. This property reflects the ability of materials to withstand stresses during mastication, being widely used to assess the mechanical performance of PMMA denture base resins, particularly via three-point bending tests that simulate clinically relevant loading conditions.^45-48^ Nevertheless, findings on the effect of NF reinforcement on this property were inconsistent, with some investigations reporting improvements^2,33,34^ and others, reductions when compared with conventional PMMA.^7,35,36^ Despite this variability, the meta-analysis showed no statistically significant differences between groups. The exploratory subgroup analysis suggested that methodological heterogeneity strongly influenced pooled estimates as excluding two studies substantially reduced heterogeneity. Importantly, most experimental groups remained above the minimum flexural strength threshold established by ISO 20795-1:2013 (65 MPa), except for the group containing 5% carbon NFs in Yamazaki, et al.^35^ (2020), which showed a 64.42 MPa value.

Regarding other mechanical properties, the available evidence was also inconsistent. Impact resistance, which is clinically relevant because impact failure is frequently associated with denture fracture,^49^ showed improvement in the study by Dai, et al.^33^ (2021), with proportional increases according to NF concentration. Hardness, which reflects the resistance of materials to permanent surface deformation and may indirectly indicate wear resistance and polymerization quality,^50-52^ showed divergent behavior. Dai, et al.^3 3^ (2021) reported increased hardness with higher NF concentrations, whereas Rangel, et al.^3 6^ (2025) observed significant reductions at specific concentrations. Similarly, Uyar, et al.^2^ (2016) showed an increase in elastic modulus, suggesting greater stiffness and lower flexibility in NF-reinforced PMMA. Fracture toughness improved in the only study that evaluated this property,^2^ suggesting enhanced resistance to crack propagation.^53-55^

Evidence regarding physical and surface-related properties was limited. Color stability, an important factor for the aesthetic longevity of denture base materials,^56^ was evaluated in only one study, which reported slight changes in L* and a* parameters following incorporation of PMMA-Ce NFs.^36^ Although these alterations made the material lighter and greener, they were clinically imperceptible.^36^ Surface roughness is another relevant property because values above 0.2 μm may favor microbial adhesion and biofilm accumulation.^57,58^ Rangel, et al.^36^ (2025) observed no significant changes in surface roughness after NF incorporation, with all values remaining within clinically acceptable limits. Contact angle, which reflects surface wettability and hydrophobicity,^59,60^ was also evaluated in only one study. Increased contact angle values were observed with increasing NF concentration, suggesting greater surface hydrophobicity.^36^ Since hydrophobic surfaces may favor biofilm formation by *Candida* spp.,^47,61,62^ this finding may have biological implications that warrant further investigation.

This systematic review has limitations that should be acknowledged. No clinical studies met the eligibility criteria, which limits direct clinical extrapolation of the findings. Methodological heterogeneity in the included studies, including differences in NF type and concentration, polymerization methods, specimen dimensions, and testing standards, compromised direct comparability and influenced pooled estimates. Moreover, some studies provided limited quantitative data reporting, restricting data extraction, and the small number of included studies precluded robust assessment of publication bias. Although exploratory subgroup analysis reduced heterogeneity, these findings should be interpreted with caution as this non-prespecified analysis involved a limited dataset. Future investigations should adopt more standardized experimental protocols and reporting methods to strengthen the evidence regarding NF reinforcement on PMMA denture base resins.

## Conclusions

Although the incorporation of NFs into PMMA has been investigated as a strategy to modify its physicomechanical properties, the current evidence remains inconsistent. Meta-analysis of flexural strength showed no statistically significant differences between the NF-reinforced groups and conventional PMMA, although the exploratory subgroup analysis suggested a reduction on this property at higher NF concentrations. Findings regarding other physicomechanical properties were also variable across studies. The lack of standardization, absence of sample size calculations, and scarcity of *in vivo* studies limit the clinical applicability of the available evidence. Therefore, further studies adopting standardized experimental protocols and well-designed *in vivo* investigations are needed to explain the effects of NF incorporation on the physicomechanical performance, durability, and biocompatibility of PMMA denture base resins.

## Data Availability

All data generated or analyzed during this study are included in this published article.
